# Supplementary material for: GP and parent dissonance about the assessment and treatment of childhood eczema in primary care: a qualitative study
Source: BMJ Open. 2018 Feb 15;8(2):e019633. doi: 10.1136/bmjopen-2017-019633 (PMC5829900; doi:10.1136/bmjopen-2017-019633)
Supplement: Supplementary Appendix 1 [file bmjopen-2017-019633supp001.pdf]

Appendix A: Coding framework

Codes and sub-codes	
1	**Practice demographics**
2	**Family set up –** Number of family members in household, number of family members with eczema, carers of children i.e. nursery, grandparents etc.
3	**How eczema started and was diagnosed**
5	**Healthcare professional (HCP)/stakeholder biography -** years in practice/specialist interest
6	**HCP knowledge about eczema**<br>Sub-codes:<br>- Frequency of seeing children with eczema<br>- How parents perceive HCPs knowledge<br>- HCP dermatology training<br>- HCP confidence/knowledge around diagnosis<br>- HCP confidence/knowledge around treatment<br>- Resources used by HCPs (GPSI/books/guidelines/websites)
7	**Eczema consultations**<br>Sub-codes:<br>- Support/advice given (including written info and signposting)<br>- Follow up arrangements<br>- Lack of HCP time (to give support/advice/education)<br>- Recurrent visits to HCP<br>- Patient confidence in HCPs<br>- History taking (this refers to the questions about the condition that GPs ask their patients during an eczema consultation)<br>- HCP attitude
8	**Medications**<br>Sub-codes:<br>- Medications prescribed (what meds are prescribed/repeat prescriptions)<br>- Assessing patient compliance with meds<br>- Parent requests for particular treatments<br>- Quantities of treatments prescribed<br>- Trial and error (this refers to where different emollients are trialled to identify patient preference for a particular type of emollient – e.g. gel, lotion, cream, ointment)<br>- HCP views about medicated ointments (i.e. topical corticosteroids)<br>- Parent views about medicated ointments<br>- Appropriate use of meds/compliance<br>- Prescribing restrictions<br>- Adverse reactions to meds<br>- Parent acceptability of treatment
9	**Complementary therapies** - complementary therapies used by parents/parent desire to use natural products
10	**Referrals**<br>Sub-codes:<br>- Referral pathways<br>- Reasons for referral<br>- HCP pressure to refer/parent desire for referral
11	**Eczema symptoms**<br>Sub-codes:<br>- Recognising flares/infection<br>- Symptoms (e.g. difficulty stopping the child scratching)

	- Different types of skin<br>- Triggers<br>- Impact of eczema
12	**Allergy/diet** - parent questions/beliefs about allergy and diet, and management of these by the HCP
13	**Parent knowledge about eczema**<br>Sub-codes:<br>- Parent knowledge<br>- Sources of knowledge (HCPs/internet/friends and family) (includes advice about treatments)<br>- Prior experience of eczema (e.g. parent or other sibling had it)<br>- HCP view of parental knowledge<br>- Support/advice desired by parents
14	**Parent beliefs about eczema**
15	**Parent guilt** – guilt about asking for more prescriptions/wasting doctors time
16	**Financial burden of eczema for patients**
17	**Problems parents face when managing the condition**<br>Sub-codes:<br>- Stress/emotions/mental health<br>- Conflicting advice/information<br>- Confusion<br>- Overload of information<br>- Need for better education<br>- Lack of support from HCPs (incl lack of continuity of care)<br>- Recall of information<br>- Access to HCP appointments<br>- Medical jargon
18	**Past experience of WAPs (Written Action Plans)**
19	**Value of WAPs**<br>Sub-codes:<br>- Benefits<br>- Which patients they are useful for<br>- Dis-benefits<br>- Timing of WAP
20	**WAP content**<br>Sub-codes:<br>- Specific patient-related content (what medication, how much, and allergies and other conditions that child has)<br>- General content (how to apply meds, signposting, hints and tips, bathing, what eczema is etc)<br>- Other content (photo/consent to share/when to seek help etc./log of treatments used)
21	**WAP format**<br>Sub-codes:<br>- Colour vs black and white<br>- Length (incl displaying on fridge)<br>- Paper vs electronic<br>- Pictures vs text<br>- Child friendly
22	**Completing the WAP**<br>- Ease of use<br>- Who should be completing it

23	**Sharing the WAP**<br>Sub-codes:<br>- Who to share it with<br>- How to share it
24	**Updating WAP**
25	**Critique of sample WAPs**
26	**Challenges of WAPs in primary care** - includes language barriers/lack of GP time to complete WAP/IT considerations/HCP familiarity with WAP
27	**Patient vs HCP perspective of eczema** – differences between parental and HCP views of eczema
28	**Managing dissonance between patient and GP views –** ways in which GPs attempted to manage areas of dissonance with parents.
29	**Resources/training need to support WAP use**
30	**Other resources to support eczema care**
