# Supplementary material for: GP and parent dissonance about the assessment and treatment of childhood eczema in primary care: a qualitative study
Source: BMJ Open. 2018 Feb 15;8(2):e019633. doi: 10.1136/bmjopen-2017-019633 (PMC5829900; doi:10.1136/bmjopen-2017-019633)
Supplement: Author's manuscript [file bmjopen-2017-019633.draft_revisions.pdf]

BMJ Open

BMJ Open is committed to open peer review. As part of this commitment we make the peer review history of every article we publish publicly available.

When an article is published we post the peer reviewers' comments and the authors' responses online. We also post the versions of the paper that were used during peer review. These are the versions that the peer review comments apply to.

The versions of the paper that follow are the versions that were submitted during the peer review process. They are not the versions of record or the final published versions. They should not be cited or distributed as the published version of this manuscript.

BMJ Open is an open access journal and the full, final, typeset and author-corrected version of record of the manuscript is available on our site with no access controls, subscription charges or pay-per-view fees (http://bmjopen.bmj.com).

If you have any questions on BMJ Open's open peer review process please email editorial.bmjopen@bmj.com

BMJ Open

GP and parent dissonance about the assessment and treatment of childhood eczema in primary care: a qualitative study

Journal:	*BMJ Open*
Manuscript ID	bmjopen-2017-019633
Article Type:	Research
Date Submitted by the Author:	15-Sep-2017
Complete List of Authors:	Powell, Kingsley; University of Bristol, Centre for Academic Primary Care<br>Le Roux, Emma; University of Bristol School of Social and Community Medicine<br>Banks, Jonathan; University of Bristol, Social & Community Medicine<br>Ridd, Matthew; University of Bristol, Social & Community Medicine; University of Bristol
**Primary Subject Heading**:	Dermatology
Secondary Subject Heading:	Paediatrics
Keywords:	Atopic dermatitis, PRIMARY CARE, QUALITATIVE RESEARCH, Management, PAEDIATRICS, Atopic eczema

SCHOLARONE™
Manuscripts

For peer review only

Title page

Title

GP and parent dissonance about the assessment and treatment of childhood eczema in primary care: a qualitative study

Authors

Kingsley Powell[1], Emma Le Roux[1], Jonathan Banks[2], Matthew J Ridd[1]

[1] Centre for Academic Primary Care, School of Social and Community Medicine, University of Bristol, Bristol.

[2]National Institute for Health Research Collaborations for Leadership in Applied Health Research and Care West (NIHR CLAHRC West), University Hospitals Bristol NHS Foundation Trust, Bristol.

Corresponding author: Matthew Ridd (m.ridd@bristol.ac.uk)

Target journal: BMJ Open

Manuscript word and table count

Including quotes: 3975

Excluding quotes: 2709

Tables: 1

For peer review only

For peer review only - http://bmjopen.bmj.com/site/about/guidelines.xhtml

Abstract

Objectives: To compare parents' and clinicians' perspectives on the assessment and treatment of children with eczema.

Design: Qualitative study

Setting: General Practices in the UK

Participants: Parents of children with eczema and General Practitioners (GPs)

Results: 11 parents and 15 GPs took part in semi-structured individual interviews. We identified several areas of dissonance between parents and GPs. First, parents sought a 'cause' of eczema, such as an underlying allergy, whereas GPs looked to manage the symptoms of an incurable condition. Second, parents often judged eczema severity in terms of psychosocial impact, while GPs tended to focus on the appearance of the child's skin. Third, parents sought 'more natural' over the counter treatments or complementary and alternative medicine, which GPs felt unable to endorse because of their unknown effectiveness and potential harm. Fourth, GPs linked poor outcomes to low unrealistic expectations of treatment and low adherence to topical therapy, whereas parents reported persisting with treatment and despondency with its ineffectiveness. Consultations were commonly described by parents as being dominated by the GP, with a lack of involvement in treatment decisions. GPs' management of divergent views varied, but avoidance strategies were often employed.

Conclusions: Divergent views between parents and clinicians regarding the aetiology and treatment of childhood eczema can probably only be bridged by clinicians actively seeking out opinions and sharing rationale for their approach to treatment. Together with assessing the psychosocial as well as the physical impact of eczema, asking about current or intended use of complementary therapy and involving parents in treatment decisions, the management of eczema and patient outcomes could be improved.

Word count: 263/300

Strengths and limitations

- First qualitative study to directly explore and compare GP and parent perspectives around eczema management
- Diverse sample of GPs and parents
- Several GPs had a personal or professional interest in eczema
- Paired interviews may have given a better understanding of areas of divergent views

For peer review only - http://bmjopen.bmj.com/site/about/guidelines.xhtml

For peer review only

Introduction

Eczema is a common childhood condition characterised by dry and itchy skin.(1) However, due to the fluctuating nature of the condition, carers must adapt their treatment regime according to the child's symptoms and the appearance of the skin. Emollients are usually used in the maintenance phase and different strength topical corticosteroids (TCS) or calcineurin inhibitors used to treat an exacerbation, or 'flare'. Consequently treatment regimens can become complex and poor adherence has been associated with poor clinical outcomes.(1)

Factors affecting treatment adherence and outcomes in childhood eczema include: the time taken to apply treatments; confusion about what, when and how to use prescribed medications;(2) and parental fear of TCS side effects.(3, 4) The doctor-patient relationship is a key factor influencing treatment outcomes.(2) Good verbal and non-verbal communication, shared decision making, practitioner interest in the patient and acknowledgement of psychosocial issues contribute to a more positive relationship which in turn can lead to improved treatment adherence.(2) Parents' views of and approach to eczema treatments may be different to those of a clinician. In the UK, most children with eczema are managed in primary care yet parents can feel that the condition is not taken seriously enough by their General Practitioner (GP).(5) However, research in this area has focused on the views and experiences of parents and children and the perspective of primary care clinicians, and particularly the interaction between GP and patient, has not been examined in any detail.

As part of a wider study to develop an eczema Written Action Plan (WAP) (6) for children we explored the experiences of managing eczema from a range of perspectives. In a previous publication, we described the clinical perspective in relation to training, diagnosis and treatment.(7) In this paper, we extend this analysis by comparing the contrasting views of GPs and parents in order to better understand the issues and their impact on the care of children with eczema.

Method

As part of the wider study,(6) we asked parents and GPs about the challenges of diagnosing and managing eczema via semi-structured interviews.

Recruitment and sampling

We interviewed GPs and parents of children under 12 years with eczema, the age group representing the majority of eczema cases in primary care.(8) Parents were recruited from five socioeconomically diverse GP surgeries by written invitation and sampled by the child's age, gender and ethnicity, and self-reported eczema severity.(9) Children were given the opportunity to participate in the interview at their parents' discretion. GPs were recruited from the same practices and through personal and professional networks. They were sampled by gender, years in job role, experience of using and perceived value of WAPs for eczema, and socio-demographic area of the practice.

Data collection

Data collection took place between May 2016 and February 2017. Interviews lasted between 45 – 60 minutes, and took place in participants' homes, on NHS premises, or by telephone. The interviewers (KP, a non-clinical researcher and ELR, a GP with a specialist interest in dermatology) used a topic guide specific for each participant group (i.e. parents and GPs) to facilitate discussion. Table 1 shows the issues covered that are relevant to the findings in this paper. Data collection ended once we achieved data saturation within the parent and GP groups with respect to the main research questions around developing a WAP.

For peer review only - http://bmjopen.bmj.com/site/about/guidelines.xhtml

Table 1: Topic guide questions around experience of managing eczema

Parents	GPs
• What difficulties have you encountered around diagnosis/ treatment?<br>• What treatments have you used?<br>• What advice/support have been given?<br>• What follow up have you received?	• What difficulties have you encountered around diagnosis/treatment?<br>• What advice do you give and what do you prescribe?<br>• What follow up arrangements do you make?<br>• How do you try and support parents in looking after their child?

Data analysis

All interview transcripts were transcribed verbatim and imported into QSR NVivo 10 software. A coding framework, as part of the constant comparative thematic analysis approach,(10) was developed and refined by KP and ELR alongside data collection and agreed with the wider study team. KP and ELR separately applied the coding framework to eight transcripts and discussed and resolved any discrepancies to maximise inter-rater coding agreement. Codes were compared within and across participant groups and then grouped into themes and sub-themes.

Results

We interviewed 11 parents (two with children), and 15 GPs. Participant characteristics are shown in Table 2. Of the doctors, 10 were GP partners and five associates; two had a specialist interest in dermatology and two declared a personal experience of eczema (family member affected). Their mean self-reported confidence in managing eczema (0 low, 10 high) was 7.3 (range 5 to 9). Participating parents' children had a mean eczema severity score of 15.3 (range = 1-27), based on the Patient Oriented Eczema Measure(9) (based on the most affected child for 10 of the 11 parents).

Parents and GPs had different perspectives on the causes, impact and treatment of the condition. We explore the divergent perspectives between parent and GP across several dimensions of primary care consultations for childhood eczema including assessment, treatment options and treatment adherence/efficacy.

Table 2: Participant characteristics

	**GPs**	**Parents**	**Child**
Mean age in years (range)	43.6 years (30-56)	37.4 years (27-48)	5.3 years (0-11)
Mean years in Role (range)	14.1 years (0.5 to 29)	-	-
Gender			
Male	4	0	4
Female	11	11	7

For peer review only - http://bmjopen.bmj.com/site/about/guidelines.xhtml

Divergent views about assessment and cause

One area of division between GPs and parents was around the assessment of the condition and its severity:

> *'I think patients' understanding of what bad eczema is often very different from ours so sometimes they say oh it's a terrible rash and, you know, it's the faintest, palest little bit of redness so because they haven't ever seen the severity of what eczema can be'* (019G:GP)

In describing the condition GPs tended to focus on the physical appearance of eczema and symptoms such as scratching and disturbed sleep. In comparison, parents emphasised the wider psychosocial impact of eczema on the family, social activities and emotional well-being:

> *'there's no way we could even contemplate camping. There's sort of limits- every single night they've got to have a bath, its- he can't have a normal life but don't get me wrong, it's not life threatening and, you know, compared with what he could have its amazing but people have no idea, they haven't got a clue the impact it has.'* (025P:parent)

Parents felt this wider impact was often overlooked/underestimated by GPs:

> *'You kind of go in and the doctor thinks oh child's a bit itching, they're not sleeping, rah rah rah, is this just a parent who is being over reactive when in reality you've got a screaming child who is crawling at their skin'* (034P:parent)

There were also differing views between parents and GPs about the cause of eczema. Parents often spoke of seeking a 'cure' and held beliefs about an underlying, remediable cause to their child's eczema, most commonly allergy, whereas GPs approached it as an incurable, long-term condition:

> *'when a child is suffering from the eczema the main thing we have to focus on what causes it. It could be for definite it was the diet, it was like allergic to food…'* (018P:parent)

> *'I don't think they find it difficult to accept that this is called eczema. I think its difficulties accepting that it will be chronic and they will have a tendency to it and accepting that's it not caused by something that they can treat and then it will go away.'* (021G:GP)

GPs spoke of the challenge of addressing these parental beliefs and resolving discrepant perspectives:

> *'I think they expect that if they can find the one trigger for the eczema they can make it all magically go away so you have to unpick that.'* (041G:GP)

Managing divergent views around allergy and complementary/alternative medicine

GPs described how in addition to managing the child's condition, they 'managed' parent views about allergy testing and non-prescribed treatments. GPs noted that parents commonly voiced concerns about allergy during consultations. The difficulty for GPs was weighing up parents' concerns against the clinical likelihood of an allergy, the poor accuracy of the tests and concern about overloading the allergy services:

> *'You take a food history and see if there are any triggers but if they can't usually identify any I don't tend to pursue that. I don't know if that's good practice or not to be honest but it's my general approach to things.'* (030G:GP)

Strategies to manage this tension varied. Some GPs spoke of acknowledging parents' beliefs (*'trying to understand what their concern is'* [017G], or taking an allergy focused history to identify a potential problem and, if not, diplomatically explaining that allergy is unlikely to be a factor. Others

For peer review only - http://bmjopen.bmj.com/site/about/guidelines.xhtml

spoke of trying to *'steer'* (031G) parents away from this idea and '*encourage them to stick to the mainstay'* (021G), i.e. controlling it with emollients:

> *'I try and explore what they think allergy testing is and what they think they will achieve by having it and ultimately when they- what they're actually asking for is a referral to a specialist which is in some cases entirely fair enough but I think as I said I think they come in armed with a lot of misinformation about it and kind of hope that it's the holy grail which you can completely understand, but, yeah, I think it's a slightly murky field'* (041G:GP)

When parents in our study had raised concerns about allergy, they were generally happy to take their GPs' advice. However, they had difficulty reconciling this advice when it conflicted with that received from other sources such as the internet and peers:

> I: *'Is there anything else that would have helped you during that time?*
>
> P: *'Yes, to get a concise answer on whether allergy testing is worth doing or not 'cos the number of people who've said to me over the years oh well when I had eczema or my, you know, seventh cousin removed had eczema then they cut out this, that and the other, if I'd followed all of that he would eat nothing at all and yet I've always been told by the medical profession we don't allergy test children with eczema because sometimes they'll react, sometimes they won't…'* (025P:parent)

One parent reported that the GP 'dismissed' their concern about allergy:

> *'My son is allergic to dairy but she [daughter] isn't but I did say to the GP could it be that she shouldn't have dairy and she said- she just dismissed that and said no but I- it's difficult isn't it? I don't know the evidence about, you know, allergies and skin conditions….if there is research about diet and eczema I'd like to know about that'* (028P:parent)

Parents understood "complementary and alternative" treatments differently. Some talked about products bought over-the-counter (OTC), such as moisturisers and coconut oil, which was common place; while others talked of herbal remedies and acupuncture, hereon referred to as CAM (Complementary and Alternative Medicine). Some parents turned to CAM in the hope of finding a cure, or in an attempt to better manage eczema symptoms, while for others it was about using more 'natural' products that they deemed to be safer for their child's skin. Parents felt these therapies were effective and they wanted recognition of this from their clinician:

> *'And also not dismissing, you know, if you are somebody that wants to use more natural products on your child not to dismiss that, you know, as- for instance I use coconut oil for her cradle cap and that's actually been amazing, you know, rather than using all these different shampoos. There are some natural things that really do work and, you know, you kind of want that holistic, you know, care for your child and just that that's accepted in the medical world.'* (028P:parent)

GPs reported that use of OTC and CAM use among parents was common and uncommon respectively, although one GP felt '*we probably only know the fraction of it*' (031G). They felt unable to endorse use of CAM or OTC products because of their unknown effectiveness and concerns about harmful additives:

> *'Yeah, people talk about lots of other products, aloe vera comes up, but it's difficult for me to assess whether- what the efficacy of those are so I tend to sort of gently, not sort of poo poo it but I gently steer them back to what I know works because I don't always know what additives or product, you know, how good they are really-'* (019G:GP)

For peer review only - http://bmjopen.bmj.com/site/about/guidelines.xhtml

'I know you're not supposed to use the Chinese things anymore 'cos they've probably got steroids in them aren't they?' (016G:GP)

GPs varied in whether they actively probed parents on their use of CAM or OTC products. One GP said she specifically asked parents, others said parents *'tend to come out with it'* (035G), whilst for others still it was not *'on the top of my list to actively ask'* (031G:GP).

Parents and GPs agreed that limited time within the consultation was a contributory factor for these issues and concerns about allergy not being addressed:

'in the ideal world, yes, me and my nurse we would be spending time explaining all of it but in the world we live in that's impossible' (015G:GP)

'you only get like ten minutes with the doctor don't you? It's very brief, and they'll say yes its eczema, here's the cream, whack it on two to three times a week and then they send you out the door but you don't know, like you say, it could be an allergy thing that started it off.' (027P:parent)

Treatment decisions, adherence and efficacy

GPs and parents reported a trial and error approach to finding an emollient that suited the family. Eczema guidelines advise healthcare professionals to offer parents and children a choice of emollients.(1, 11, 12) Whilst several GPs cited the importance of patient preference, their prescribing decisions around emollients seemed to be based on their own preferences or their experiences with other parents, rather than involving parents on an individual basis:

'So I tend to use quite a lot of Hydromol ointment 'cos from my own experience of my daughter found it really good…' (035G:GP)

This mirrored the parents' experiences of encounters with GPs, where their role in emollient choice was often depicted as passive compared to the directive manner of the GPs:

'…the GP does his thing and I'm off with my big jars of moisturisers…' (033P:parent)

'…they're like oh it's just eczema, like you know chuck that at you and I, like I said, that's the second dose now he's had of that one…' (014P:parent)

Poor treatment adherence in primary care was commonly reported among GPs. With TCS, they said it was related to parental fear of their side effects, i.e. skin thinning, which was corroborated by the parents themselves. With emollients, some GPs felt that it was a result of parents having unrealistic expectations:

'they just want it fixed now, they're not thinking to keep on applying, they'll use it short term and then it'll be ok and then they'll come back.' (029G:GP)

'I think its difficulties accepting that it will be chronic and they will have a tendency to it and accepting that's it not caused by something that they can treat and then it will go away.' (021G:GP)

Conversely, many parents reported persisting with topical therapy, but declared feelings of desperation and frustration at the futility of topical treatments due to their apparent inefficacy:

'It just kept on getting worse and worse. I've used everything. I've used Oilatum, Aveeno, I've used derma- is it Dermatol or something like that for the head. I've used steroid creams but then I've just stopped using steroids because I looked at whenever they go- if it goes it

For peer review only - http://bmjopen.bmj.com/site/about/guidelines.xhtml

comes back when you stop and it's not really advised to use it for a long time anyway so what's the point?' (033P:parent)

Parents also described feelings of guilt about requesting more emollient from their GP, or felt that requests were met with resistance:

> *'Sometimes I have to fight my corner. I've now got it so that he gets- we get through 500 grams of hydromol a day so at one point people would only give me enough to last barely a week. I've now got it so that we pick up sort of ten kilos a time. Yeah, sometimes- depending on who's doing the repeat prescriptions you sometimes have to fight your corner a bit and I have to make sure I write a long letter to my GP if we're going away for longer than a week or two to explain why I need ridiculous amounts of everything'* (025P:parent)

> *'I feel a bit guilty asking for more prescriptions at the doctors (laughs) they must think oh I only did that, you know, a month ago or whatever.'* (028P:parent)

Despondency with topical therapy appeared to relate to parents' lower confidence in clinicians who did not have specialist skills/knowledge. They described more confidence in dermatologists and GPs with a specialist interest or training in dermatology as they felt they gave more credible advice, and treatments that 'worked':

> *'for credibility, to believe what they tell- what they tell me, what they tell me works and I'm going to follow it I would want it to be someone who is trained by dermatology or Eczema Society and does it frequently for other families.'* (000P:parent)

> *'I think she just gave me a bit more information really and I trusted her experience of what is actually going to work … and she explained that eczema quite frequently gets infected and it would be better to use that (Dermol) regularly as her moisturiser'* (28P:parent)

Discussion

Summary

We identified several areas of divergent views between parents and GPs regarding disease aetiology, assessment and treatment. First, parents looked for a 'cure' for eczema, often fixated on the role of allergy. Clinicians, on the other hand, were more focused on just 'managing' the condition. Second, some parents wanted clinicians to provide a more holistic approach to assessment and treatment, and to acknowledge the role of complementary or non-prescribed therapies. However, clinicians did not regularly enquire about use of non-prescribed treatments and were not willing to support their use because of uncertainty about their provenance and absence of evidence regarding their efficacy or safety. Third, parents perceived GPs as dismissive and felt they did not recognise the full impact of eczema on the child's quality of life. GPs felt that poor response to treatment was a result of lack of adherence to emollients which was linked to parents wanting a quick 'fix'. However, parents reported generally persisting with topical therapy but often felt it was ineffective. Finally, parents described GP-parent interactions as being dominated by the doctor, and the lack of parental involvement in treatment decisions was apparent in data from both perspectives.

Strengths and limitations

To our knowledge this is the first qualitative study to directly explore and compare GP and parent perspectives on eczema management and treatment. Our sample gave us a diverse range of views from parents (with respect to age, ethnicity of child and their self-rated eczema severity score) and GPs (with respect to age, years of working and sociodemographic population of the practice). However, GPs were predominantly female/GP partners and several had a professional or personal

For peer review only - http://bmjopen.bmj.com/site/about/guidelines.xhtml

interest in eczema meaning the views captured may not be representative of all GPs. Despite this, our GP participants appeared to be unaware of the importance parents place on the wider impact of eczema, e.g. the emotional and psychosocial elements. While some of the GPs and parents came from the same practice, the interviews were not 'paired'. Paired interviews may have added to the richness of the data and our understanding of areas of divergence. The data may also have been influenced by the status of the interviewer: the majority of interviews with GPs were carried out by a GP researcher with a specialist interest in dermatology and the parent interviews by a non-clinical researcher.(13) Data gathered from the two children interviewed alongside their parents are not presented because they did not contribute any information around GP-patient dissonance.

Comparison with other literature

Patient resistance to topical treatments; perceived GP disinterest in eczema and its psychological impact; concerns about GP competency; and parental pursuit of allergy as cause and a 'cure' have been previously described.(5, 14, 15) Gore et al (16) outlined parents' wide ranging and complex informational needs, including the requirement to address concerns about aetiology and any role for diet. However, we also found differences in parents' and GPs' approaches to assessing eczema, their views of the reasons for barriers to effective emollient therapy and their perspectives around CAM use.

Parents of children with eczema often want an 'active' role in treatment decision making in order to address a perceived lack of (or inconsistent) information and the lack of time and/or interest from health professionals.(16) Patients' agendas are commonly not voiced which can lead to misunderstanding.(17) Showing empathy and asking questions about the impact of the condition can positively affect the relationship between parent and GP,(2, 14) and is in line with current clinical guidance which advocates a holistic approach to eczema assessment.(1, 11) However, our study shows that even when GPs are aware of disparate views, for example parent beliefs around allergy, it does not necessarily result in discussion and resolution of these beliefs. Rather, GPs described a tendency to avoid rather than confront them, with concerns about the access to, and interpretation of, allergy tests cited as factors.

Previous research suggests that CAM use is common among eczema patients.(18, 19) Despite the risks attached to use of some forms of CAM,(20) it is promoted as being effective and safe, (21) and is used by patients looking for 'natural' treatments and a 'cure',(19, 22) both of which were reasons for its use by parents in our study. Clinical guidelines encourage open discussions between healthcare professionals and parents about CAM treatments for eczema and explanations about the lack of evidence for its safety and efficacy.(1, 11) However, patients rarely disclose CAM use (23) and healthcare professionals rarely ask about it.(24, 25) Similarly in our study, conversations about CAM were not high on the GPs' agenda, which may partly be due to their perception that it is not commonly used for eczema.

Another reported reason for CAM use is patient dissatisfaction with conventional eczema treatments,(19) where parents sometimes feel emollients do not 'work'.(5) This was reflected in our interviews with parents where they persisted with topical therapy but often felt it was ineffective. However, emollients are widely accepted in the medical world as the mainstay for eczema management (26). Emollients come in different formulations, but there is limited evidence to support the use of one emollient over another, therefore patient preference is a key part of prescribing decisions.(27) Although the GPs in our study recognised the importance of patient preference, they talked about prescribing emollients based on their own or other parents' preferences, rather than on an individual basis.

For peer review only - http://bmjopen.bmj.com/site/about/guidelines.xhtml

Implications for research and practice

Good eczema care requires a significant degree of self-management but engagement in such processes needs to be built on a solid foundation of understanding around causes and treatment between GP and parent. Rather than shying away from these conversations, clinicians should be prepared to engage in these discussions and explain the rationale behind their approach. Longer, initial consultations may facilitate this, although this must be accompanied by skills and knowledge that builds parental trust.

Clinicians are reminded that assessments of disease severity should include questions about psychosocial impact as well as a physical examination. Better shared decision making, particularly around treatment preferences, may also improve treatment adherence. As evidence to guide emollient choice is lacking,(28) healthcare professionals and parents need to openly discuss individual needs and preferences to find a treatment that is both suitable and acceptable for the family. Treatment discussions should also include current or intended use of CAM as parents may be unaware of its potential risks. Understanding why a parent is using CAM, for example because they are dissatisfied with their emollient, may inform future prescribing decisions.

Our work, and that of others, could be extended through observation of consultations, to assess how these divergent perspectives are played out in practice and how they might be addressed. In addition to supporting GP undergraduate and postgraduate dermatology education, research is needed to find ways to support consultations in primary care for eczema. The written action plan we have developed as part of the wider project may be one way of facilitating these discussions.(6)

Contributorship statement

KP is the main author, leading on data collection, analysis and write-up. ELR was also involved in data collection, analysis and contributed to revisions of the write-up. MR obtained funding for the study and MR/JB were involved in the methodological design and contributed to analysis via discussions during regular research meetings, as well as having significant input to drafts of the written paper.

Funding

This work/MR's time was supported by the National Institute for Health Research Post-Doctoral Research Fellowship (PDF-2014-07-013). The views expressed in this publication are those of the authors and not necessarily those of the NHS, the National Institute for Health Research or the Department of Health.

KP was funded by NIHR Research Capability Funding from the Avon Primary Care Research Collaborative on behalf of Bristol, North Somerset and South Gloucestershire CCGs.

ELR's time was supported by Elizabeth Blackwell Institute for Health Research, University of Bristol and the Wellcome Trust Institutional Strategic Support Fund.

JB's time was supported by the National Institute for Health Research (NIHR) Collaboration for Leadership in Applied Health Research and Care West (CLAHRC West) at University Hospitals Bristol NHS Foundation Trust.

Ethical approval

The study received ethical approval from the Yorkshire and the Humber – Bradford Leeds Research Ethics Committee (REC reference 16/YH/0179).

For peer review only - http://bmjopen.bmj.com/site/about/guidelines.xhtml

Competing interests

The authors have declared no competing interests.

Acknowledgements

We would like to thank all the participants who participated in this research, the West of England Clinical Research Network, the UK Dermatology Clinical Trials Network, and the practices who were involved in facilitating this study, and the members of the Patient and Public Involvement and Engagement group. Thanks also to Dr Alice Malpass, University of Bristol, who commented on the study protocol, and Nancy Horlick, University of Bristol, who transcribed the audio files.

Data sharing statement

Participants in this study consented to other researchers using the anonymised data to support future research, the data of this study being used to promote scientific knowledge and for no other purpose than research. Therefore, for bona fide researchers, the fully anonymised dataset has been deposited at the University of Bristol Research Data Repository (http://data.bris.ac.uk). A metadata record is openly available by the repository, with a link (data-bris@bristol.ac.uk) to the Research Data team at Bristol who provide information on how data can be accessed by bona fide researchers, and who will assess the motives of potential data re-users before granting access to the data. No authentic request for access will be refused and re-users will not be charged for any part of this process.

References

1. NICE clinical guidance 57. Atopic eczema in children. 2007.
2. Sokolova A, Smith SD. Factors contributing to poor treatment outcomes in childhood atopic dermatitis. Australasian Journal of Dermatology. 2015;56(4):252-7.
3. Bewley A, Dermatology Working G. Expert consensus: time for a change in the way we advise our patients to use topical corticosteroids. British Journal of Dermatology. 2008;158(5):917-20.
4. Charman CR, Morris AD, Williams HC. Topical corticosteroid phobia in patients with atopic eczema. British Journal of Dermatology. 2000;142(5):931-6.
5. Santer M, Burgess H, Yardley L, Ersser S, Lewis-Jones S, Muller I, et al. Experiences of carers managing childhood eczema and their views on its treatment: a qualitative study. British Journal of General Practice. 2012;62(597):e261-e7.
6. Powell K, Le Roux E, Banks J, Ridd MJ. Developing a written action plan for children with eczema: a qualitative study. British Journal of General Practice.In press.
7. Le Roux E, Powell K, Banks J, Ridd MJ. GPs' experiences of diagnosing and managing childhood eczema: a qualitative study. Submitted.
8. NICE clinical guidance 57: Atopic eczema in children costing report. 2007.
9. Charman C, Venn A, Ravenscroft J, Williams H. Translating Patient-Oriented Eczema Measure (POEM) scores into clinical practice by suggesting severity strata derived using anchor-based methods. British Journal of Dermatology. 2013;169(6):1326-32.
10. Glaser B. The Constant Comparative Method of Qualitative Analysis. The Discovery of Grounded Theory: Strategies for Qualitative Research. Edited by: Glaser B, Strauss A. 1967. Chicago: ALDINE Publishing Company.
11. Scottish Intercollegiate Guidelines Network. Management of atopic eczema in primary care: a national clinical guideline, (2011).
12. Eichenfield LF, Tom WL, Berger TG, Krol A, Paller AS, Schwarzenberger K, et al. Guidelines of care for the management of atopic dermatitis: section 2. Management and treatment of atopic

For peer review only - http://bmjopen.bmj.com/site/about/guidelines.xhtml

dermatitis with topical therapies. Journal of the American Academy of Dermatology. 2014;71(1):116-32.
13. Richards H, Emslie C. The 'doctor'or the 'girl from the University'? Considering the influence of professional roles on qualitative interviewing. Family practice. 2000;17(1):71-5.
14. Magin P, Adams J, Heading G, Pond C. Patients with skin disease and their relationships with their doctors: a qualitative study of patients with acne, psoriasis and eczema. Medical Journal of Australia. 2009.
15. Noerreslet M, Jemec GB, Traulsen JM. Involuntary autonomy: patients' perceptions of physicians, conventional medicines and risks in the management of atopic dermatitis. Social Science & Medicine. 2009;69(9):1409-15.
16. Gore C, Johnson R, Caress A, Woodcock A, Custovic A. The information needs and preferred roles in treatment decision-making of parents caring for infants with atopic dermatitis: a qualitative study. Allergy. 2005;60(7):938-43.
17. Barry CA, Bradley CP, Britten N, Stevenson FA, Barber N. Patients' unvoiced agendas in general practice consultations: qualitative study. BMJ. 2000;320(7244):1246-50.
18. Jean D, Cyr C. Use of complementary and alternative medicine in a general pediatric clinic. Pediatrics. 2007;120(1):e138-e41.
19. Hughes R, Ward D, Tobin AM, Keegan K, Kirby B. The Use of Alternative Medicine in Pediatric Patients with Atopic Dermatitis. Pediatric Dermatology. 2007;24(2):118-20.
20. Niggemann B, Grüber C. Side-effects of complementary and alternative medicine. Allergy. 2003;58(8):707-16.
21. Ernst E, Pittler MH, Stevinson C. Complementary/Alternative Medicine in Dermatology. American Journal of Clinical Dermatology. 2002;3(5):341-8.
22. Magin PJ, Adams J, Heading GS, Pond DC, Smith W. Complementary and alternative medicine therapies in acne, psoriasis, and atopic eczema: results of a qualitative study of patients' experiences and perceptions. Journal of Alternative & Complementary Medicine. 2006;12(5):451-7.
23. Robinson A, McGrail M. Disclosure of CAM use to medical practitioners: a review of qualitative and quantitative studies. Complementary therapies in medicine. 2004;12(2):90-8.
24. Flannery MA, Love MM, Pearce KA, Luan JJ, Elder WG. Communication about complementary and alternative medicine: perspectives of primary care clinicians. Alternative Therapies in Health and Medicine. 2006;12(1):56.
25. Robinson N, Lorenc A, Falinski A, Banarsee R. The challenges of facilitating primary healthcare discussions on traditional, complementary and alternative medicine for childhood eczema: Piloting a computerized template. Patient Education and Counseling. 2012;89(3):517-24.
26. Lewis-Jones S, Cork M, Clark C. Atopic eczema in children–guideline consultation: a systematic review of the treatments for atopic eczema and guideline for its management. London: National Institute for Clinical Excellence (NICE). 2007.
27. Lawton S. Practical issues for emollient therapy in dry and itchy skin. British Journal of Nursing. 2009;18(16).
28. EJ vZ, Z F, R C, A L, BWM A. Emollients and moisturisers for eczema Cochrane Database of Systematic Reviews. 2017(2).

For peer review only - http://bmjopen.bmj.com/site/about/guidelines.xhtml

COREQ Statement

GP and parent dissonance about the assessment and treatment of childhood eczema in primary care: a qualitative study

Domain 1: Research team and reflexivity

Personal Characteristics

1. Interviewer/facilitator. Which author/s conducted the interview or focus group?

a. Kingsley Powell (KP) – please see page 3 (Data collection)

b. Emma Le Roux (ELR) – please see page 3 (Data collection)

2. Credentials. What were the researcher's credentials? E.g. PhD, MD.

a. MSc

b. MBBS, MRCGP, DPD

3. Occupation. What was their occupation at the time of the study?

a. Research Associate

b. Elizabeth Blackwell Institute Clinical Primer Research Fellow

4. Gender. Was the researcher male or female?

Both female

5. Experience and training. What experience or training did the researcher have?

KP has had training in qualitative research methods and conducting qualitative research at undergraduate and postgraduate level (BSc & MSc), and attended a 'Writing a qualitative paper' course (School of Social and Community Medicine, 30.11.2016) and an 'Introduction to NVivo' workshop (School of Social and Community Medicine, 08.12.2016).

ELR underwent training through attendance at an 'Introduction to Qualitative Interviewing' course: University of Surrey (1day; 8.9.2016) as well as completing a Masters in Primary Care Research module on Qualitative research methods (Sept-Dec 2016; Manchester University) and attending a 'Writing a qualitative paper' course: School of Social and Community Medicine, Bristol University (1 day; 30.11.16) and NVIVO workshop: IT training, University of Bristol (1day; 8.11.16).

Relationship with participants

6. Relationship established. Was a relationship established prior to study commencement?

ELR knew 5 GP interviewees professionally.

7. Participant knowledge of the interviewer. What did the participants know about the researcher? e.g. personal goals, reasons for doing the research?

Each interviewee was sent an information sheet explaining the reasons for conducting the research. The reasons for the study were explained prior to each individual interview. KP had no personal goals or reasons for doing the research. ELR with her specialist interest in Dermatology was interested in understanding the experiences and challenges GPs face when managing childhood eczema.

8. Interviewer characteristics. What characteristics were reported about the interviewer/facilitator? e.g. Bias, assumptions, reasons and interests in the research topic.

For peer review only

KP is a non-clinical researcher and ELR is a clinical researcher (GP with a dermatology interest). Both declared their academic standpoint to interviewees so that they knew whether the interviewer was from a medical background or not.

Domain 2: study design

Theoretical framework

9. Methodological orientation and Theory. What methodological orientation was stated to underpin the study? e.g. grounded theory, discourse analysis, ethnography, phenomenology, content analysis

In the Methods/ Data Analysis section we explain how we used semi-structured interviews and thematic analysis, informed by the constant comparison method. Please see page 4, Data Analysis.

Participant selection

10. Sampling. How were participants selected? e.g. purposive, convenience, consecutive, snowball.

Sampling for all participants, parents and GPs, was purposive, See page 3, Recruitment and Sampling and also point 16 below.

11. Method of approach. How were participants approached? e.g. face-to-face, telephone, mail, email

Parents (by letter in the post) and GPs (by email) were invited to register their interest in taking part in the study by completing an online survey which was then used to inform sampling. At this initial contact participants were given a copy of the participant information sheet for the study. Interested parents/clinicians were then sampled and those selected to take part by the study team were contacted by phone or email, asked if they were still willing to take part and, if yes, a face-to-face or telephone interview was arranged. At this, and all stages of recruitment, participants were invited to ask questions about the study.

12. Sample size. How many participants were in the study?

We recruited 11 parents of children with eczema and 15 GPs. Please see page 4, Results.

13. Non-participation. How many people refused to participate or dropped out? Reasons

53 parents expressed interest in taking part, 19 were invited to interview (plus one parent from our local Patient and Public Involvement and Engagement network), and 11 took part, with 8 parents either declining or not responding to our attempts at contact. 48 GPs expressed interest in taking part, 32 of those were invited to interview, and 15 took part, with 17 either declining or not responding to our attempts at contact.

Setting

14. Setting of data collection. Where was the data collected? e.g. home, clinic, workplace

Data from parents were mainly collected at the participants' homes, but one parent interview was conducted over the telephone due to geographical reasons (they had recently moved from the area and were no longer local). Data from GPs were mainly collected in GP practices. Five interviews were conducted in GPs' homes and two over the telephone.

15. Presence of non-participants. Was anyone else present besides the participants and researchers?

No, except in two cases where the child with eczema also took part in the interview along with their parent after reading an information sheet and signing an assent form.

For peer review only - http://bmjopen.bmj.com/site/about/guidelines.xhtml

16. Description of sample. What are the important characteristics of the sample? e.g. demographic data, date.
GPs were sampled by gender, years in job role, experience of using and perceived value of WAPs for eczema. Parents were sampled by the child's age, gender and ethnicity, and self-reported eczema severity.

Data collection
17. Interview guide. Were questions, prompts, guides provided by the authors? Was it pilot tested?
A separate, semi-structured topic guide with questions and prompts was used for each participant group, i.e. parents and GPs. The focus of the topic guide for the wider study was around the experiences of managing eczema and the role of a written action plan, and what a written action plan should like and contain. The topic guide was piloted with a parent from a local Patient and Public Involvement and Engagement group and modifications were made.

18. Repeat interviews.
We did not conduct repeat interviews

19. Audio/visual recording. Did the research use audio or visual recording to collect the data?
Yes, audio recording was used throughout data collection.

20. Field notes. Were field notes made during and/or after the interview or focus group?
Yes, summary and reflective/reflexive notes were made during and after each interview.

21. Duration. What was the duration of the interviews or focus group?
The interviews lasted between 20 and 68 minutes.

22. Data saturation. Was data saturation discussed?
Yes this was discussed in team meetings.

23. Transcripts returned. Were transcripts returned to participants for comment and/or correction?
No, it was not possible to offer in the time available in the study.

Domain 3: analysis and findings
Data analysis
24. Number of data coders. How many data coders coded the data?
KP and ELR independently coded the same 8 transcripts and then compared codes and discussed discrepancies to achieve a coding consensus and maximise rigour. Codes were also agreed with the wider study team. Please see Page 4, Data Analysis.

25. Description of the coding tree. Did authors provide a description of the coding tree?
No.

26. Derivation of themes. Were themes identified in advance or derived from the data?
Themes were derived inductively from the data.

27. Software. What software, if applicable, was used to manage the data?
We used NVivo 10 qualitative software package to manage the data.

28. Participant checking. Did participants provide feedback on the findings?

For peer review only

No.

29. Quotations presented. Were participant quotations presented to illustrate the themes / findings? Was each quotation identified? e.g. participant number
Yes unique participant codes were used alongside quotes, to illustrate findings. The roles of the interviewees (i.e. parent or GP) were identified within the codes.

30. Data and findings consistent. Was there consistency between the data presented and the findings?
Yes

31. Clarity of major themes. Were major themes clearly presented in the findings?
Yes

32. Clarity of minor themes. Is there a description of diverse cases or discussion of minor themes?
Yes, we present contrasting GP approaches to managing patients' questions about allergy, and the variation among GPs about actively asking about use of complementary and alternative medicine/over the counter products.

For peer review only

BMJ Open

GP and parent dissonance about the assessment and treatment of childhood eczema in primary care: a qualitative study

Journal:	*BMJ Open*
Manuscript ID	bmjopen-2017-019633.R1
Article Type:	Research
Date Submitted by the Author:	30-Nov-2017
Complete List of Authors:	Powell, Kingsley; University of Bristol, Centre for Academic Primary Care<br>Le Roux, Emma; University of Bristol School of Social and Community Medicine<br>Banks, Jonathan; University of Bristol, Social & Community Medicine<br>Ridd, Matthew; University of Bristol, Social & Community Medicine; University of Bristol
**Primary Subject Heading**:	Dermatology
Secondary Subject Heading:	Paediatrics
Keywords:	Atopic dermatitis, PRIMARY CARE, QUALITATIVE RESEARCH, Management, PAEDIATRICS, Atopic eczema

SCHOLARONE™
Manuscripts

For peer review only

Title page

Title

GP and parent dissonance about the assessment and treatment of childhood eczema in primary care: a qualitative study

Authors

Kingsley Powell[1], Emma Le Roux[1], Jonathan Banks[2], Matthew J Ridd[1]

[1] Centre for Academic Primary Care, School of Social and Community Medicine, University of Bristol, Bristol.

[2]National Institute for Health Research Collaborations for Leadership in Applied Health Research and Care West (NIHR CLAHRC West), University Hospitals Bristol NHS Foundation Trust, Bristol.

Corresponding author: Matthew Ridd (m.ridd@bristol.ac.uk)

Target journal: BMJ Open

Manuscript word and table count

Including quotes: 4395

Excluding quotes: 3129

Tables: 2

For peer review only - http://bmjopen.bmj.com/site/about/guidelines.xhtml

Abstract

Objectives: To compare parents' and clinicians' perspectives on the assessment and treatment of children with eczema in primary care.

Design: Qualitative interview study with purposive and snowballing sampling and thematic analysis.

Setting: 14 General Practices in the UK

Participants: 11 parents of children with eczema and 15 General Practitioners (GPs) took part in semi-structured individual interviews.

Results: We identified several areas of dissonance between parents and GPs. First, parents sought a 'cause' of eczema, such as an underlying allergy, whereas GPs looked to manage the symptoms of an incurable condition. Second, parents often judged eczema severity in terms of psychosocial impact, while GPs tended to focus on the appearance of the child's skin. Third, parents sought 'more natural' over the counter treatments or complementary and alternative medicine, which GPs felt unable to endorse because of their unknown effectiveness and potential harm. Fourth, GPs linked poor outcomes to unrealistic expectations of treatment and low adherence to topical therapy, whereas parents reported persisting with treatment and despondency with its ineffectiveness. Consultations were commonly described by parents as being dominated by the GP, with a lack of involvement in treatment decisions. GPs' management of divergent views varied, but avoidance strategies were often employed.

Conclusions: Divergent views between parents and clinicians regarding the cause and treatment of childhood eczema can probably only be bridged by clinicians actively seeking out opinions and sharing rationale for their approach to treatment. Together with assessing the psychosocial as well as the physical impact of eczema, asking about current or intended use of complementary therapy and involving parents in treatment decisions, the management of eczema and patient outcomes could be improved.

Word count: 272/300

Strengths and limitations

- First qualitative study to directly explore and compare GP and parent perspectives around eczema management
- Diverse sample of GPs and parents
- Several GPs had a personal or professional interest in eczema
- Paired interviews may have given a better understanding of areas of divergent views
- As secondary findings to the main research questions, these themes require further exploration.

For peer review only - http://bmjopen.bmj.com/site/about/guidelines.xhtml

Introduction

Eczema is a common childhood condition characterised by dry and itchy skin.(1) However, due to the fluctuating nature of the condition, carers must adapt their treatment regime according to the child's symptoms and the appearance of the skin. Emollients are usually used in the maintenance phase and different strength topical corticosteroids (TCS) or calcineurin inhibitors used to treat an exacerbation, or 'flare'. Consequently treatment regimens can become complex and poor adherence has been associated with poor clinical outcomes.(1)

Factors affecting treatment adherence and outcomes in childhood eczema include: the time taken to apply treatments; confusion about what, when and how to use prescribed medications;(2) and parental fear of TCS side effects.(3, 4) The doctor-patient relationship is a key factor influencing treatment outcomes.(2) Good verbal and non-verbal communication, shared decision making, practitioner interest in the patient and acknowledgement of psychosocial issues contribute to a more positive relationship which in turn can lead to improved treatment adherence.(2) Parents' views of and approach to eczema treatments may be different to those of a clinician. In the UK, most children with eczema are managed in primary care yet parents can feel that the condition is not taken seriously enough by their General Practitioner (GP).(5) However, research in this area has focused on the views and experiences of parents and children and the perspective of primary care clinicians, and particularly the interaction between GP and patient, has not been examined in any detail.

As part of a wider study to develop an eczema Written Action Plan (WAP) (6) for children we explored the experiences of managing eczema from a range of perspectives. In a previous publication, we described the clinical perspective in relation to training, diagnosis and treatment.(7) In this paper, to better understand the issues and their impact on the care of children with eczema more generally, we extend this analysis by comparing for the first time the contrasting views of GPs and parents.

Method

Qualitative interview study to identify the experience of managing childhood eczema in primary care from the perspective of GPs and parents.

Recruitment and sampling

We interviewed GPs and parents of children under 12 years with eczema, the age group representing the majority of eczema cases in primary care.(8) Parents were recruited from five socioeconomically diverse GP surgeries by written invitation and sampled by the child's age, gender and ethnicity, and self-reported eczema severity.(9) Children were given the opportunity to participate in the interview at their parents' discretion. GPs were recruited from the same practices and through personal and professional networks. They were sampled by gender, years in job role, experience of using and perceived value of WAPs for eczema, and socio-demographic area of the practice. Parents' index of multiple deprivation (IMD) were obtained using their home postcode.(10)

Data collection

Data collection took place between May 2016 and February 2017. All participants gave written informed consent to take part in the study. Interviews lasted typically between 45 – 60 minutes, and took place in participants' homes, on NHS premises, or by telephone. The interviewers (KP, a non-clinical researcher and ELR, a GP with a specialist interest in dermatology) used a topic guide specific for each participant group (i.e. parents and GPs) to facilitate discussion. The initial versions were based on the findings of a qualitative and quantitative literature review of self-management/WAPs

For peer review only - http://bmjopen.bmj.com/site/about/guidelines.xhtml

in children with long-term conditions/eczema.(11, 12) In discussion with the research team, they were refined iteratively over the course of the study, as new areas warranting further exploration emerged. Table 1 shows the issues covered that are relevant to the findings in this paper. ELR interviewed most of the GPs (13/15) and KP interviewed all of the parents. To minimise any biases, both researchers wrote reflective and reflexive accounts throughout the interviews and we discussed any issues with the study team, made up of both clinical and non-clinical researchers, at regular team meetings. KP and ELR also independently double-coded a sample of the transcripts (see below). Data collection ended once we achieved data saturation within the parent and GP groups with respect to the main research questions around developing a WAP. Whilst the main research question was focused on the WAP development, this was underpinned by identifying issues that parents and children face in primary care consultations and therefore the findings presented in this paper were a key element of our data collection.

Table 1: Topic guide questions around experience of managing eczema

Parents	GPs
• What difficulties have you encountered around diagnosis/ treatment?<br>• What treatments have you used?<br>• What advice/support have been given?<br>• What follow up have you received?	• What difficulties have you encountered around diagnosis/treatment?<br>• What advice do you give and what do you prescribe?<br>• What follow up arrangements do you make?<br>• How do you try and support parents in looking after their child?

Data analysis

All interview transcripts were transcribed verbatim and imported into QSR NVivo 10 software. A coding framework, as part of the constant comparative thematic analysis approach,(13) was developed and refined by KP and ELR alongside data collection and agreed with the wider study team. KP and ELR separately applied the coding framework (Appendix A) to eight transcripts and discussed and resolved any discrepancies to maximise inter-rater coding agreement. Themes were developed by line by line analyses and coding of the data which led to clear patterns in the data from both parents and GPs. Codes were compared within and across participant groups and then grouped into themes and sub-themes.

Patient and public involvement

Between two and four parents met three times in 1.5-2 hour small group meetings over the duration of the broader study. As part of the development of the eczema WAP, they were invited to comment on the findings. They reported that the “barriers to treatment” described in this paper had strong face validity.

Results

We interviewed 11 parents (two with children), and 15 GPs. Participant characteristics are shown in Table 2. Of the doctors, 10 were GP partners and five associates; two had a specialist interest in dermatology and two declared a personal experience of eczema (family member affected). Their mean self-reported confidence in managing eczema (0 low, 10 high) was 7.3 (range 5 to 9).

For peer review only - http://bmjopen.bmj.com/site/about/guidelines.xhtml

Participating parents' children had a mean eczema severity score of 15.3 (range = 1-27), based on the Patient Oriented Eczema Measure(9) (based on the most affected child for 10 of the 11 parents). Parents had an mean IMD of 5.2 (range 1-9; 1, low, 10 high).

Parents and GPs had different perspectives on the causes, impact and treatment of the condition. We explore the divergent perspectives between parent and GP across several dimensions of primary care consultations for childhood eczema including assessment, treatment options and treatment adherence/efficacy.

Table 2: Participant characteristics

	**GPs**	**Parents**	**Child**
Mean age in years (range)	43.6 years (30-56)	37.4 years (27-48)	5.3 years (0-11)
Mean years in Role (range)	14.1 years (0.5 to 29)	-	-
Gender			
Male	4	0	4
Female	11	11	7

Divergent views about assessment and cause

One area of division between GPs and parents was around the assessment of the condition and its severity:

> *'I think patients' understanding of what bad eczema is often very different from ours so sometimes they say oh it's a terrible rash and, you know, it's the faintest, palest little bit of redness so because they haven't ever seen the severity of what eczema can be'* (019G:GP)

In describing the condition GPs focused more on the physical appearance of eczema and symptoms such as scratching and disturbed sleep. In comparison, parents emphasised the wider psychosocial impact of eczema on the family, social activities and emotional well-being:

> *'there's no way we could even contemplate camping. There's sort of limits- every single night they've got to have a bath, its- he can't have a normal life but don't get me wrong, it's not life threatening and, you know, compared with what he could have its amazing but people have no idea, they haven't got a clue the impact it has.'* (025P:parent)

Parents felt this wider impact was often overlooked/underestimated by GPs:

> *'You kind of go in and the doctor thinks oh child's a bit itching, they're not sleeping, rah rah rah, is this just a parent who is being over reactive when in reality you've got a screaming child who is crawling at their skin'* (034P:parent)

There were also differing views between parents and GPs about the cause of eczema. Parents often spoke of seeking a 'cure' and held beliefs about an underlying, remediable cause to their child's eczema, most commonly allergy, whereas GPs approached it as an incurable, long-term condition:

> *'when a child is suffering from the eczema the main thing we have to focus on what causes it. It could be for definite it was the diet, it was like allergic to food…'* (018P:parent)

For peer review only

For peer review only - http://bmjopen.bmj.com/site/about/guidelines.xhtml

> *'I don't think they find it difficult to accept that this is called eczema. I think its difficulties accepting that it will be chronic and they will have a tendency to it and accepting that's it not caused by something that they can treat and then it will go away.'* (021G:GP)

GPs spoke of the challenge of addressing these parental beliefs and resolving discrepant perspectives:

> *'I think they expect that if they can find the one trigger for the eczema they can make it all magically go away so you have to unpick that.'* (041G:GP)

Managing divergent views around allergy and complementary/alternative medicine

GPs described how in addition to managing the child's condition, they 'managed' parent views about allergy testing and non-prescribed treatments. GPs noted that parents commonly voiced concerns about allergy during consultations. The difficulty for GPs was weighing up parents' concerns against the clinical likelihood of an allergy, the poor accuracy of the tests and concern about overloading the allergy services:

> *'You take a food history and see if there are any triggers but if they can't usually identify any I don't tend to pursue that. I don't know if that's good practice or not to be honest but it's my general approach to things.'* (030G:GP)

Strategies to manage this tension varied. Some GPs spoke of acknowledging parents' beliefs (*'trying to understand what their concern is'* [017G], or taking an allergy focused history to identify a potential problem and, if not, diplomatically explaining that allergy is unlikely to be a factor. Others spoke of trying to *'steer'* (031G) parents away from this idea and '*encourage them to stick to the mainstay'* (021G), i.e. controlling it with emollients:

> *'I try and explore what they think allergy testing is and what they think they will achieve by having it and ultimately when they- what they're actually asking for is a referral to a specialist which is in some cases entirely fair enough but I think as I said I think they come in armed with a lot of misinformation about it and kind of hope that it's the holy grail which you can completely understand, but, yeah, I think it's a slightly murky field'* (041G:GP)

When parents in our study had raised concerns about allergy, they said they were happy to take their GPs' advice. However, they had difficulty reconciling this advice when it conflicted with that received from other sources such as the internet and peers:

> I: *'Is there anything else that would have helped you during that time?*
>
> P: *'Yes, to get a concise answer on whether allergy testing is worth doing or not 'cos the number of people who've said to me over the years oh well when I had eczema or my, you know, seventh cousin removed had eczema then they cut out this, that and the other, if I'd followed all of that he would eat nothing at all and yet I've always been told by the medical profession we don't allergy test children with eczema because sometimes they'll react, sometimes they won't…'* (025P:parent)

One parent reported that the GP 'dismissed' their concern about allergy:

> *'My son is allergic to dairy but she [daughter] isn't but I did say to the GP could it be that she shouldn't have dairy and she said- she just dismissed that and said no but I- it's difficult isn't it? I don't know the evidence about, you know, allergies and skin conditions….if there is research about diet and eczema I'd like to know about that'* (028P:parent)

For peer review only

For peer review only - http://bmjopen.bmj.com/site/about/guidelines.xhtml

Parents understood "complementary and alternative" treatments differently. Some talked about products bought over-the-counter (OTC), such as moisturisers and coconut oil, which was common place; while others talked of herbal remedies and acupuncture, hereon referred to as CAM (Complementary and Alternative Medicine). Some parents turned to CAM in the hope of finding a cure, or in an attempt to better manage eczema symptoms, while for others it was about using more 'natural' products that they deemed to be safer for their child's skin. Parents felt these therapies were effective and they wanted recognition of this from their clinician:

> *'And also not dismissing, you know, if you are somebody that wants to use more natural products on your child not to dismiss that, you know, as- for instance I use coconut oil for her cradle cap and that's actually been amazing, you know, rather than using all these different shampoos. There are some natural things that really do work and, you know, you kind of want that holistic, you know, care for your child and just that that's accepted in the medical world.'* (028P:parent)

GPs reported that use of OTC and CAM use among parents was common and uncommon respectively, although one GP felt '*we probably only know the fraction of it*' (031G). They felt unable to endorse use of CAM or OTC products because of their unknown effectiveness and concerns about harmful additives:

> *'Yeah, people talk about lots of other products, aloe vera comes up, but it's difficult for me to assess whether- what the efficacy of those are so I tend to sort of gently, not sort of poo poo it but I gently steer them back to what I know works because I don't always know what additives or product, you know, how good they are really-'* (019G:GP)

> *'I know you're not supposed to use the Chinese things anymore 'cos they've probably got steroids in them aren't they?'* (016G:GP)

GPs varied in whether they actively probed parents on their use of CAM or OTC products. One GP said she specifically asked parents, others said parents *'tend to come out with it'* (035G), whilst for others still it was not *'on the top of my list to actively ask'* (031G:GP).

Parents and GPs agreed that limited time within the consultation was a contributory factor for these issues and concerns about allergy not being addressed:

> *'in the ideal world, yes, me and my nurse we would be spending time explaining all of it but in the world we live in that's impossible'* (015G:GP)

> *'you only get like ten minutes with the doctor don't you? It's very brief, and they'll say yes its eczema, here's the cream, whack it on two to three times a week and then they send you out the door but you don't know, like you say, it could be an allergy thing that started it off.'* (027P:parent)

Treatment decisions, adherence and efficacy

GPs and parents reported a trial and error approach to finding an emollient that suited the family. Eczema guidelines advise healthcare professionals to offer parents and children a choice of emollients.(1, 14, 15) Whilst several GPs cited the importance of patient preference, their prescribing decisions around emollients seemed to be based on their own preferences or their experiences with other parents, rather than involving parents on an individual basis:

> *'So I tend to use quite a lot of Hydromol ointment 'cos from my own experience of my daughter found it really good…'* (035G:GP)

For peer review only - http://bmjopen.bmj.com/site/about/guidelines.xhtml

This mirrored the parents' experiences of encounters with GPs, where their role in emollient choice was often depicted as passive compared to the directive manner of the GPs:

> *'…the GP does his thing and I'm off with my big jars of moisturisers…'* (033P:parent)

> *'…they're like oh it's just eczema, like you know chuck that at you and I, like I said, that's the second dose now he's had of that one…'* (014P:parent)

Poor treatment adherence in primary care was commonly reported among GPs. With TCS, they said it was related to parental fear of their side effects, i.e. skin thinning, which was corroborated by the parents themselves. With emollients, some GPs felt that it was a result of parents having unrealistic expectations:

> *'they just want it fixed now, they're not thinking to keep on applying, they'll use it short term and then it'll be ok and then they'll come back.'* (029G:GP)

> *'I think its difficulties accepting that it will be chronic and they will have a tendency to it and accepting that's it not caused by something that they can treat and then it will go away.'* (021G:GP)

Conversely, many parents reported persisting with topical therapy, but declared feelings of desperation and frustration at the futility of topical treatments due to their apparent inefficacy:

> *'It just kept on getting worse and worse. I've used everything. I've used Oilatum, Aveeno, I've used derma- is it Dermatol or something like that for the head. I've used steroid creams but then I've just stopped using steroids because I looked at whenever they go- if it goes it comes back when you stop and it's not really advised to use it for a long time anyway so what's the point?'* (033P:parent)

Parents also described feelings of guilt about requesting more emollient from their GP, or said that requests were met with resistance:

> *'Sometimes I have to fight my corner. I've now got it so that he gets- we get through 500 grams of hydromol a day so at one point people would only give me enough to last barely a week. I've now got it so that we pick up sort of ten kilos a time. Yeah, sometimes- depending on who's doing the repeat prescriptions you sometimes have to fight your corner a bit and I have to make sure I write a long letter to my GP if we're going away for longer than a week or two to explain why I need ridiculous amounts of everything'* (025P:parent)

> *'I feel a bit guilty asking for more prescriptions at the doctors (laughs) they must think oh I only did that, you know, a month ago or whatever.'* (028P:parent)

Despondency with topical therapy appeared to relate to parents' lower confidence in clinicians who did not have specialist skills/knowledge. They described more confidence in dermatologists and GPs with a specialist interest or training in dermatology as they felt they gave more credible advice, and treatments that 'worked':

> *'for credibility, to believe what they tell- what they tell me, what they tell me works and I'm going to follow it I would want it to be someone who is trained by dermatology or Eczema Society and does it frequently for other families.'* (000P:parent)

> *'I think she just gave me a bit more information really and I trusted her experience of what is actually going to work … and she explained that eczema quite frequently gets infected and it would be better to use that (Dermol) regularly as her moisturiser'* (28P:parent)

For peer review only

For peer review only - http://bmjopen.bmj.com/site/about/guidelines.xhtml

Discussion

Summary

We identified several areas of divergent views between parents and GPs regarding disease aetiology, assessment and treatment. First, parents looked for a 'cure' for eczema, often fixated on the role of allergy. Clinicians, on the other hand, were more focused on just 'managing' the condition. Second, some parents wanted clinicians to provide a more holistic approach to assessment and treatment, and to acknowledge the role of complementary or non-prescribed therapies. However, clinicians did not regularly enquire about use of non-prescribed treatments and were not willing to support their use because of uncertainty about their provenance and absence of evidence regarding their efficacy or safety. Third, parents perceived GPs as dismissive and felt they did not recognise the full impact of eczema on the child's quality of life. GPs felt that poor response to treatment was a result of lack of adherence to emollients, which was linked to parents wanting a quick 'fix'. However, parents reported generally persisting with topical therapy but often felt it was ineffective. Finally, parents described GP-parent interactions as being dominated by the doctor, and the lack of parental involvement in treatment decisions was apparent in data from both perspectives.

Strengths and limitations

To our knowledge this is the first qualitative study to directly explore and compare GP and parent perspectives on eczema management and treatment. Our sample gave us a diverse range of views from parents (with respect to age, ethnicity of child and their self-rated eczema severity score) and GPs (with respect to age, years of working and sociodemographic population of the practice). However, GPs were predominantly female/GP partners and several had a professional or personal interest in eczema meaning the views captured may not be representative of all GPs. Despite this, our GP participants appeared to be unaware of the importance parents place on the wider impact of eczema, e.g. the emotional and psychosocial elements. While some of the GPs and parents came from the same practice, the interviews were not 'paired'. Paired interviews may have added to the richness of the data and our understanding of areas of divergence. The data may also have been influenced by the status of the interviewer: the majority of interviews with GPs were carried out by a GP researcher with a specialist interest in dermatology and the parent interviews by a non-clinical researcher.(16) Data gathered from the two children interviewed alongside their parents are not presented because they did not contribute any information around GP-patient dissonance.

Comparison with other literature

Patient resistance to topical treatments; perceived GP disinterest in eczema and its psychological impact; concerns about GP competency; and parental pursuit of allergy as cause and a 'cure' have been previously described.(5, 17, 18) Gore et al (19) outlined parents' wide ranging and complex informational needs, including the requirement to address concerns about aetiology and any role for diet. However, we also found differences in parents' and GPs' approaches to assessing eczema, their views of the reasons for barriers to effective emollient therapy and their perspectives around CAM use.

Parents of children with eczema often want an 'active' role in treatment decision making in order to address a perceived lack of (or inconsistent) information and the lack of time and/or interest from health professionals.(19) Patients' agendas are commonly not voiced which can lead to misunderstanding.(20) Showing empathy and asking questions about the impact of the condition can positively affect the relationship between parent and GP,(2, 17) and is in line with current clinical guidance which advocates a holistic approach to eczema assessment.(1, 14) However, our study shows that even when GPs are aware of disparate views, for example parent beliefs around allergy,

For peer review only - http://bmjopen.bmj.com/site/about/guidelines.xhtml

it does not necessarily result in discussion and resolution of these beliefs. Rather, GPs described a tendency to avoid rather than confront them, with concerns about the access to, and interpretation of, allergy tests cited as factors.

Previous research suggests that CAM use is common among eczema patients.(21, 22) Despite the risks attached to use of some forms of CAM,(23) it is promoted as being effective and safe, (24) and is used by patients looking for 'natural' treatments and a 'cure',(22, 25) both of which were reasons for its use by parents in our study. Clinical guidelines encourage open discussions between healthcare professionals and parents about CAM treatments for eczema and explanations about the lack of evidence for its safety and efficacy.(1, 14) However, patients rarely disclose CAM use (26) and healthcare professionals rarely ask about it.(27, 28) Similarly in our study, conversations about CAM were not high on the GPs' agenda, which may partly be due to their perception that it is not commonly used for eczema.

Another reported reason for CAM use is patient dissatisfaction with conventional eczema treatments,(22) where parents sometimes feel emollients do not 'work'.(5) This was reflected in our interviews with parents where they persisted with topical therapy but often felt it was ineffective. Santer et al found that parents had mixed views about long-term use of emollients to prevent flare-ups (29) and this was echoed by the GPs in our study who said that parents were not using emollients for long enough to be effective and were expecting results with short-term use. Education about the rationale for emollients may lead to more positive parental attitudes towards long-term use.(29) Emollients are widely accepted in the medical world as the mainstay for eczema management in that they are the first line of treatment even when the skin is clear (30). They come in different formulations, but there is limited evidence to support the use of one emollient over another, therefore patient preference is a key part of prescribing decisions.(31) However, the other key treatment in primary care for children with all but the mildest eczema is appropriate use of topical corticosteroids, concerns about which can be a significant barrier to achieving and maintaining disease control.(5, 32)

Implications for research and practice

Good eczema care requires a significant degree of self-management but engagement in such processes needs to be built on a solid foundation of understanding around causes and treatment between GP and parent. Rather than shying away from these conversations, clinicians should be prepared to engage in these discussions and explain the rationale behind their approach. Longer, initial consultations may facilitate this, although this must be accompanied by skills and knowledge that builds parental trust. There is some evidence that nurse led clinics may improve eczema control which could be used to support time-limited GPs, although evidence of benefit in a primary care setting is lacking.(11)

Clinicians are reminded that assessments of disease severity should include questions about psychosocial impact as well as a physical examination. Better shared-decision making, particularly around treatment preferences, together with signposting of reliable sources of information may also improve treatment adherence. As evidence to guide emollient choice and the long-term safety of topical corticosteroids is lacking,(33) healthcare professionals and parents need to openly discuss individual needs and preferences to find a treatment that is both suitable and acceptable for the family. Treatment discussions should also include current or intended use of CAM as parents may be unaware of its potential risks. Understanding why a parent is using CAM, for example because they are dissatisfied with their emollient, may inform future prescribing decisions.

For peer review only - http://bmjopen.bmj.com/site/about/guidelines.xhtml

Our work, and that of others, could be extended through observation of consultations, to assess how these divergent perspectives are played out in practice and how they might be addressed. In addition to supporting GP undergraduate and postgraduate dermatology education, research is needed to find ways to support consultations in primary care for eczema. The written action plan we have developed as part of the wider project may be one way of facilitating these discussions.(6)

Contributorship statement

KP is the main author, leading on data collection, analysis and write-up. ELR was also involved in data collection, analysis and contributed to revisions of the write-up. MR obtained funding for the study and MR/JB were involved in the methodological design and contributed to analysis via discussions during regular research meetings, as well as having significant input to drafts of the written paper.

Funding

This work/MR's time was supported by the National Institute for Health Research Post-Doctoral Research Fellowship (PDF-2014-07-013). The views expressed in this publication are those of the authors and not necessarily those of the NHS, the National Institute for Health Research or the Department of Health.

KP was funded by NIHR Research Capability Funding from the Avon Primary Care Research Collaborative on behalf of Bristol, North Somerset and South Gloucestershire CCGs.

ELR's time was supported by Elizabeth Blackwell Institute for Health Research, University of Bristol and the Wellcome Trust Institutional Strategic Support Fund.

JB's time was supported by the National Institute for Health Research (NIHR) Collaboration for Leadership in Applied Health Research and Care West (CLAHRC West) at University Hospitals Bristol NHS Foundation Trust.

Ethical approval

The study received ethical approval from the Yorkshire and the Humber – Bradford Leeds Research Ethics Committee (REC reference 16/YH/0179).

Competing interests

The authors have declared no competing interests.

Acknowledgements

We would like to thank all the participants who participated in this research, the West of England Clinical Research Network, the UK Dermatology Clinical Trials Network, and the practices who were involved in facilitating this study, and the members of the Patient and Public Involvement and Engagement group. Thanks also to Dr Alice Malpass, University of Bristol, who commented on the study protocol; Nancy Horlick, University of Bristol, who transcribed the audio files; and to the peer reviewers of this paper for their thoughtful comments.

Data sharing statement

Participants in this study consented to other researchers using the anonymised data to support future research, the data of this study being used to promote scientific knowledge and for no other purpose than research. Therefore, for bona fide researchers, the fully anonymised dataset has been deposited at the University of Bristol Research Data Repository (http://data.bris.ac.uk). A metadata

For peer review only

For peer review only - http://bmjopen.bmj.com/site/about/guidelines.xhtml

record is openly available by the repository, with a link (data-bris@bristol.ac.uk) to the Research Data team at Bristol who provide information on how data can be accessed by bona fide researchers, and who will assess the motives of potential data re-users before granting access to the data. No authentic request for access will be refused and re-users will not be charged for any part of this process.

References

1. NICE clinical guidance 57. Atopic eczema in children. 2007.
2. Sokolova A, Smith SD. Factors contributing to poor treatment outcomes in childhood atopic dermatitis. Australasian Journal of Dermatology. 2015;56(4):252-7.
3. Bewley A, Dermatology Working G. Expert consensus: time for a change in the way we advise our patients to use topical corticosteroids. British Journal of Dermatology. 2008;158(5):917-20.
4. Charman CR, Morris AD, Williams HC. Topical corticosteroid phobia in patients with atopic eczema. British Journal of Dermatology. 2000;142(5):931-6.
5. Santer M, Burgess H, Yardley L, Ersser S, Lewis-Jones S, Muller I, et al. Experiences of carers managing childhood eczema and their views on its treatment: a qualitative study. British Journal of General Practice. 2012;62(597):e261-e7.
6. Powell K, Le Roux E, Banks J, Ridd MJ. Developing a written action plan for children with eczema: a qualitative study. British Journal of General Practice.In press.
7. Le Roux E, Powell K, Banks J, Ridd MJ. GPs' experiences of diagnosing and managing childhood eczema: a qualitative study. In press.
8. NICE clinical guidance 57: Atopic eczema in children costing report. 2007.
9. Charman C, Venn A, Ravenscroft J, Williams H. Translating Patient-Oriented Eczema Measure (POEM) scores into clinical practice by suggesting severity strata derived using anchor-based methods. British Journal of Dermatology. 2013;169(6):1326-32.
10. Department for Communities and Local Government. English indices of deprivation 2015 [internet]. 2015 [accessed 24 November 2017]. Available from: http://imd-by-postcode.opendatacommunities.org/
11. Ridd MJ, King AJL, Le Roux E, Waldecker A, Huntley AL. Systematic review of self-management interventions for people with eczema. British Journal of Dermatology. 2017;177:719-34. DOI: 10.1111/bjd.15601
12. Waldecker A, Malpass A, King AJL, Ridd MJ. Written action plans for children with long-term conditions: a systematic review and synthesis of qualitative data. Health Expectations. In press. DOI: 10.1111/hex.12643.
13. Glaser B. The Constant Comparative Method of Qualitative Analysis. The Discovery of Grounded Theory: Strategies for Qualitative Research. Edited by: Glaser B, Strauss A. 1967. Chicago: ALDINE Publishing Company.
14. Management of atopic eczema in primary care: a national clinical guideline, (2011).
15. Eichenfield LF, Tom WL, Berger TG, Krol A, Paller AS, Schwarzenberger K, et al. Guidelines of care for the management of atopic dermatitis: section 2. Management and treatment of atopic dermatitis with topical therapies. Journal of the American Academy of Dermatology. 2014;71(1):116-32.
16. Richards H, Emslie C. The 'doctor'or the 'girl from the University'? Considering the influence of professional roles on qualitative interviewing. Family practice. 2000;17(1):71-5.
17. Magin P, Adams J, Heading G, Pond C. Patients with skin disease and their relationships with their doctors: a qualitative study of patients with acne, psoriasis and eczema. Medical Journal of Australia. 2009;190(2):62-4.

For peer review only - http://bmjopen.bmj.com/site/about/guidelines.xhtml

18. Noerreslet M, Jemec GB, Traulsen JM. Involuntary autonomy: patients' perceptions of physicians, conventional medicines and risks in the management of atopic dermatitis. Social Science & Medicine. 2009;69(9):1409-15.
19. Gore C, Johnson R, Caress A, Woodcock A, Custovic A. The information needs and preferred roles in treatment decision-making of parents caring for infants with atopic dermatitis: a qualitative study. Allergy. 2005;60(7):938-43.
20. Barry CA, Bradley CP, Britten N, Stevenson FA, Barber N. Patients' unvoiced agendas in general practice consultations: qualitative study. BMJ. 2000;320(7244):1246-50.
21. Jean D, Cyr C. Use of complementary and alternative medicine in a general pediatric clinic. Pediatrics. 2007;120(1):e138-e41.
22. Hughes R, Ward D, Tobin AM, Keegan K, Kirby B. The Use of Alternative Medicine in Pediatric Patients with Atopic Dermatitis. Pediatric Dermatology. 2007;24(2):118-20.
23. Niggemann B, Grüber C. Side-effects of complementary and alternative medicine. Allergy. 2003;58(8):707-16.
24. Ernst E, Pittler MH, Stevinson C. Complementary/Alternative Medicine in Dermatology. American Journal of Clinical Dermatology. 2002;3(5):341-8.
25. Magin PJ, Adams J, Heading GS, Pond DC, Smith W. Complementary and alternative medicine therapies in acne, psoriasis, and atopic eczema: results of a qualitative study of patients' experiences and perceptions. Journal of Alternative & Complementary Medicine. 2006;12(5):451-7.
26. Robinson A, McGrail M. Disclosure of CAM use to medical practitioners: a review of qualitative and quantitative studies. Complementary therapies in medicine. 2004;12(2):90-8.
27. Flannery MA, Love MM, Pearce KA, Luan JJ, Elder WG. Communication about complementary and alternative medicine: perspectives of primary care clinicians. Alternative Therapies in Health and Medicine. 2006;12(1):56.
28. Robinson N, Lorenc A, Falinski A, Banarsee R. The challenges of facilitating primary healthcare discussions on traditional, complementary and alternative medicine for childhood eczema: Piloting a computerized template. Patient Education and Counseling. 2012;89(3):517-24.
29. Santer M, Muller I, Yardley L, Lewis-Jones S, Ersser S, Little P. Parents' and carers' views about emollients for childhood eczema: qualitative interview study. BMJ Open. 2016;6(8).
30. Lewis-Jones S, Cork M, Clark C. Atopic eczema in children–guideline consultation: a systematic review of the treatments for atopic eczema and guideline for its management. London: National Institute for Clinical Excellence (NICE). 2007.
31. Lawton S. Practical issues for emollient therapy in dry and itchy skin. British Journal of Nursing. 2009;18(16).
32. Smith SD, Hong E, Fearns S, Blaszczynski A, Fischer G. Corticosteroid phobia and other confounders in the treatment of childhood atopic dermatitis explored using parent focus groups. Australasian Journal of Dermatology. 2010;51(3):168-74.
33. van Zuuren EJ, Fedorowicz Z, Christensen R, Lavrijsen A, Arents BWM. Emollients and moisturisers for eczema. Cochrane Database of Systematic Reviews. 2017(2).

For peer review only - http://bmjopen.bmj.com/site/about/guidelines.xhtml

Appendix A: Coding framework

	Codes and sub-codes
1	**Practice demographics**
2	**Family set up –** Number of family members in household, number of family members with eczema, carers of children i.e. nursery, grandparents etc.
3	**How eczema started and was diagnosed**
5	**Healthcare professional (HCP)/stakeholder biography -** years in practice/specialist interest
6	**HCP knowledge about eczema**<br>Sub-codes:<br>- Frequency of seeing children with eczema<br>- How parents perceive HCPs knowledge<br>- HCP dermatology training<br>- HCP confidence/knowledge around diagnosis<br>- HCP confidence/knowledge around treatment<br>- Resources used by HCPs (GPSI/books/guidelines/websites)
7	**Eczema consultations**<br>Sub-codes:<br>- Support/advice given (including written info and signposting)<br>- Follow up arrangements<br>- Lack of HCP time (to give support/advice/education)<br>- Recurrent visits to HCP<br>- Patient confidence in HCPs<br>- History taking (this refers to the questions about the condition that GPs ask their patients during an eczema consultation)<br>- HCP attitude
8	**Medications**<br>Sub-codes:<br>- Medications prescribed (what meds are prescribed/repeat prescriptions)<br>- Assessing patient compliance with meds<br>- Parent requests for particular treatments<br>- Quantities of treatments prescribed<br>- Trial and error (this refers to where different emollients are trialled to identify patient preference for a particular type of emollient – e.g. gel, lotion, cream, ointment)<br>- HCP views about medicated ointments (i.e. topical corticosteroids)<br>- Parent views about medicated ointments<br>- Appropriate use of meds/compliance<br>- Prescribing restrictions<br>- Adverse reactions to meds<br>- Parent acceptability of treatment
9	**Complementary therapies** - complementary therapies used by parents/parent desire to use natural products
10	**Referrals**<br>Sub-codes:<br>- Referral pathways<br>- Reasons for referral<br>- HCP pressure to refer/parent desire for referral
11	**Eczema symptoms**<br>Sub-codes:<br>- Recognising flares/infection<br>- Symptoms (e.g. difficulty stopping the child scratching)

For peer review only

	- Different types of skin<br>- Triggers<br>- Impact of eczema
12	**Allergy/diet** - parent questions/beliefs about allergy and diet, and management of these by the HCP
13	**Parent knowledge about eczema**<br>Sub-codes:<br>- Parent knowledge<br>- Sources of knowledge (HCPs/internet/friends and family) (includes advice about treatments)<br>- Prior experience of eczema (e.g. parent or other sibling had it)<br>- HCP view of parental knowledge<br>- Support/advice desired by parents
14	**Parent beliefs about eczema**
15	**Parent guilt** – guilt about asking for more prescriptions/wasting doctors time
16	**Financial burden of eczema for patients**
17	**Problems parents face when managing the condition**<br>Sub-codes:<br>- Stress/emotions/mental health<br>- Conflicting advice/information<br>- Confusion<br>- Overload of information<br>- Need for better education<br>- Lack of support from HCPs (incl lack of continuity of care)<br>- Recall of information<br>- Access to HCP appointments<br>- Medical jargon
18	**Past experience of WAPs (Written Action Plans)**
19	**Value of WAPs**<br>Sub-codes:<br>- Benefits<br>- Which patients they are useful for<br>- Dis-benefits<br>- Timing of WAP
20	**WAP content**<br>Sub-codes:<br>- Specific patient-related content (what medication, how much, and allergies and other conditions that child has)<br>- General content (how to apply meds, signposting, hints and tips, bathing, what eczema is etc)<br>- Other content (photo/consent to share/when to seek help etc./log of treatments used)
21	**WAP format**<br>Sub-codes:<br>- Colour vs black and white<br>- Length (incl displaying on fridge)<br>- Paper vs electronic<br>- Pictures vs text<br>- Child friendly
22	**Completing the WAP**<br>- Ease of use<br>- Who should be completing it

For peer review only

23	**Sharing the WAP**<br>Sub-codes:<br>- Who to share it with<br>- How to share it
24	**Updating WAP**
25	**Critique of sample WAPs**
26	**Challenges of WAPs in primary care** - includes language barriers/lack of GP time to complete WAP/IT considerations/HCP familiarity with WAP
27	**Patient vs HCP perspective of eczema** – differences between parental and HCP views of eczema
28	**Managing dissonance between patient and GP views –** ways in which GPs attempted to manage areas of dissonance with parents.
29	**Resources/training need to support WAP use**
30	**Other resources to support eczema care**

For peer review only

COREQ Statement

GP and parent dissonance about the assessment and treatment of childhood eczema in primary care: a qualitative study

Domain 1: Research team and reflexivity

Personal Characteristics

1. Interviewer/facilitator. Which author/s conducted the interview or focus group?

a. Kingsley Powell (KP) – please see page 3 (Data collection)

b. Emma Le Roux (ELR) – please see page 3 (Data collection)

2. Credentials. What were the researcher's credentials? E.g. PhD, MD.

a. MSc

b. MBBS, MRCGP, DPD

3. Occupation. What was their occupation at the time of the study?

a. Research Associate

b. Elizabeth Blackwell Institute Clinical Primer Research Fellow

4. Gender. Was the researcher male or female?

Both female

5. Experience and training. What experience or training did the researcher have?

KP has had training in qualitative research methods and conducting qualitative research at undergraduate and postgraduate level (BSc & MSc), and attended a 'Writing a qualitative paper' course (School of Social and Community Medicine, 30.11.2016) and an 'Introduction to NVivo' workshop (School of Social and Community Medicine, 08.12.2016).

ELR underwent training through attendance at an 'Introduction to Qualitative Interviewing' course: University of Surrey (1day; 8.9.2016) as well as completing a Masters in Primary Care Research module on Qualitative research methods (Sept-Dec 2016; Manchester University) and attending a 'Writing a qualitative paper' course: School of Social and Community Medicine, Bristol University (1 day; 30.11.16) and NVIVO workshop: IT training, University of Bristol (1day; 8.11.16).

Relationship with participants

6. Relationship established. Was a relationship established prior to study commencement?

ELR knew 5 GP interviewees professionally.

7. Participant knowledge of the interviewer. What did the participants know about the researcher? e.g. personal goals, reasons for doing the research?

Each interviewee was sent an information sheet explaining the reasons for conducting the research. The reasons for the study were explained prior to each individual interview. KP had no personal goals or reasons for doing the research. ELR with her specialist interest in Dermatology was interested in understanding the experiences and challenges GPs face when managing childhood eczema.

8. Interviewer characteristics. What characteristics were reported about the interviewer/facilitator? e.g. Bias, assumptions, reasons and interests in the research topic.

KP is a non-clinical researcher and ELR is a clinical researcher (GP with a dermatology interest). Both declared their academic standpoint to interviewees so that they knew whether the interviewer was from a medical background or not.

Domain 2: study design

Theoretical framework

9. Methodological orientation and Theory. What methodological orientation was stated to underpin the study? e.g. grounded theory, discourse analysis, ethnography, phenomenology, content analysis

In the Methods/ Data Analysis section we explain how we used semi-structured interviews and thematic analysis, informed by the constant comparison method. Please see page 4, Data Analysis.

Participant selection

10. Sampling. How were participants selected? e.g. purposive, convenience, consecutive, snowball.

Sampling for all participants, parents and GPs, was purposive, See page 3, Recruitment and Sampling and also point 16 below.

11. Method of approach. How were participants approached? e.g. face-to-face, telephone, mail, email

Parents (by letter in the post) and GPs (by email) were invited to register their interest in taking part in the study by completing an online survey which was then used to inform sampling. At this initial contact participants were given a copy of the participant information sheet for the study. Interested parents/clinicians were then sampled and those selected to take part by the study team were contacted by phone or email, asked if they were still willing to take part and, if yes, a face-to-face or telephone interview was arranged. At this, and all stages of recruitment, participants were invited to ask questions about the study.

12. Sample size. How many participants were in the study?

We recruited 11 parents of children with eczema and 15 GPs. Please see page 4, Results.

13. Non-participation. How many people refused to participate or dropped out? Reasons

511 parents were sent a letter from their GP asking them to participate of which 53 parents expressed interest in taking part, 19 were invited to interview (plus one parent from our local Patient and Public Involvement and Engagement network), and 11 took part, with 8 parents either declining or not responding to our attempts at contact. 48 GPs expressed interest in taking part, 32 of those were invited to interview, and 15 took part, with 17 either declining or not responding to our attempts at contact.

Setting

14. Setting of data collection. Where was the data collected? e.g. home, clinic, workplace

Data from parents were mainly collected at the participants' homes, but one parent interview was conducted over the telephone due to geographical reasons (they had recently moved from the area and were no longer local). Data from GPs were mainly collected in GP practices. Five interviews were conducted in GPs' homes and two over the telephone.

15. Presence of non-participants. Was anyone else present besides the participants and researchers?

No, except in two cases where the child with eczema also took part in the interview along with their parent after reading an information sheet and signing an assent form.

For peer review only

16. Description of sample. What are the important characteristics of the sample? e.g. demographic data, date.
GPs were sampled by gender, years in job role, experience of using and perceived value of WAPs for eczema. Parents were sampled by the child's age, gender and ethnicity, and self-reported eczema severity.

Data collection
17. Interview guide. Were questions, prompts, guides provided by the authors? Was it pilot tested?
A separate, semi-structured topic guide with questions and prompts was used for each participant group, i.e. parents and GPs. The focus of the topic guide for the wider study was around the experiences of managing eczema and the role of a written action plan, and what a written action plan should like and contain. The topic guide was piloted with a parent from a local Patient and Public Involvement and Engagement group and modifications were made.

18. Repeat interviews.
We did not conduct repeat interviews

19. Audio/visual recording. Did the research use audio or visual recording to collect the data?
Yes, audio recording was used throughout data collection.

20. Field notes. Were field notes made during and/or after the interview or focus group?
Yes, summary and reflective/reflexive notes were made during and after each interview.

21. Duration. What was the duration of the interviews or focus group?
The interviews lasted between 20 and 68 minutes.

22. Data saturation. Was data saturation discussed?
Yes this was discussed in team meetings.

23. Transcripts returned. Were transcripts returned to participants for comment and/or correction?
No, it was not possible to offer in the time available in the study.

Domain 3: analysis and findings
Data analysis
24. Number of data coders. How many data coders coded the data?
KP and ELR independently coded the same 8 transcripts and then compared codes and discussed discrepancies to achieve a coding consensus and maximise rigour. Codes were also agreed with the wider study team. Please see Page 4, Data Analysis.

25. Description of the coding tree. Did authors provide a description of the coding tree?
No.

26. Derivation of themes. Were themes identified in advance or derived from the data?
Themes were derived inductively from the data.

27. Software. What software, if applicable, was used to manage the data?
We used NVivo 10 qualitative software package to manage the data.

For peer review only

28. Participant checking. Did participants provide feedback on the findings?
No.

29. Quotations presented. Were participant quotations presented to illustrate the themes / findings? Was each quotation identified? e.g. participant number
Yes unique participant codes were used alongside quotes, to illustrate findings. The roles of the interviewees (i.e. parent or GP) were identified within the codes.

30. Data and findings consistent. Was there consistency between the data presented and the findings?
Yes

31. Clarity of major themes. Were major themes clearly presented in the findings?
Yes

32. Clarity of minor themes. Is there a description of diverse cases or discussion of minor themes?
Yes, we present contrasting GP approaches to managing patients' questions about allergy, and the variation among GPs about actively asking about use of complementary and alternative medicine/over the counter products.

For peer review only
